# Draft Genome Sequences of Pelagimyophage Mosig EXVC030M and Pelagipodophage Lederberg EXVC029P, Isolated from Devil’s Hole, Bermuda

**DOI:** 10.1128/MRA.01325-20

**Published:** 2021-02-18

**Authors:** Holger H. Buchholz, Michelle Michelsen, Rachel J. Parsons, Nicholas R. Bates, Ben Temperton

**Affiliations:** a School of Biosciences, University of Exeter, Exeter, United Kingdom; b Bermuda Institute of Ocean Sciences, St. George’s, Bermuda; c School of Ocean and Earth Sciences, Waterfront Campus, University of Southampton, Southampton, United Kingdom; Loyola University Chicago

## Abstract

We present the genomes of two isolated bacteriophages infecting Pelagibacter ubique HTCC1062. *Pelagibacter* phage Mosig EXVC030M (*Myoviridae*) and *Pelagibacter* phage Lederberg EXVC029P (*Podoviridae*) were isolated by dilution-to-extinction culturing from the oxygen minimum zone at Devil’s Hole (Harrington Sound, Bermuda).

## ANNOUNCEMENT

Viruses infecting the heterotrophic bacterial clade of *Pelagibacterales* are an important component of marine microbial communities throughout global oceans (1). Since the discovery and first isolation of four pelagiphages in 2013 (2), 38 more have been isolated and sequenced (3–5). Out of the 38 isolated pelagiphages, 36 belong to the *Podoviridae* family, with only one species each of *Myoviridae* and *Siphoviridae*. Here, we report the draft genome sequences of a novel pelagimyophage and a novel pelagipodophage, both isolated on Pelagibacter ubique HTCC1062.

A 2-liter water sample was taken (12 July 2019) using a hand-held Niskin bottle, fired at a 20-m depth at Devil’s Hole, Bermuda, a seasonal oxygen minimum zone in Bermuda (6) (latitude 32.32421, longitude −64.71849). The water sample was taken to the Bermuda Institute of Ocean Sciences for processing, where planktonic cells were removed with 0.1-μm polyethersulfone filters. Viruses were concentrated by tangential flow filtration (50R VivaFlow 100-kDa Hydrosart filter; Sartorius Lab Instruments, Göttingen, Germany). We used previously described dilution-to-extinction-based methods (4) with HTCC1062 as a bait host (grown in artificial seawater medium ASM1 [7]) in 96-well Teflon plates, which does not rely on plaque formation, because the host does not grow on solid medium. The purification process was repeated five times; nonetheless, final sequence data contained two genomes, suggesting an impure culture.

For DNA isolation, a 50-ml HTCC1062 culture (10^6^ cells/ml), amended with 5 ml of 0.1-μm-filtered lysate, was grown in ASM1 (18°C) until cell death (detected via flow cytometry). Debris was removed using 0.1-μm-pore polyvinylidene difluoride (PVDF) filters, and lysate was subjected to PEG8000/NaCl DNA isolation (modified from https://doi.org/10.17504/protocols.io.c36yrd, as described previously (4).

**FIG 1:**
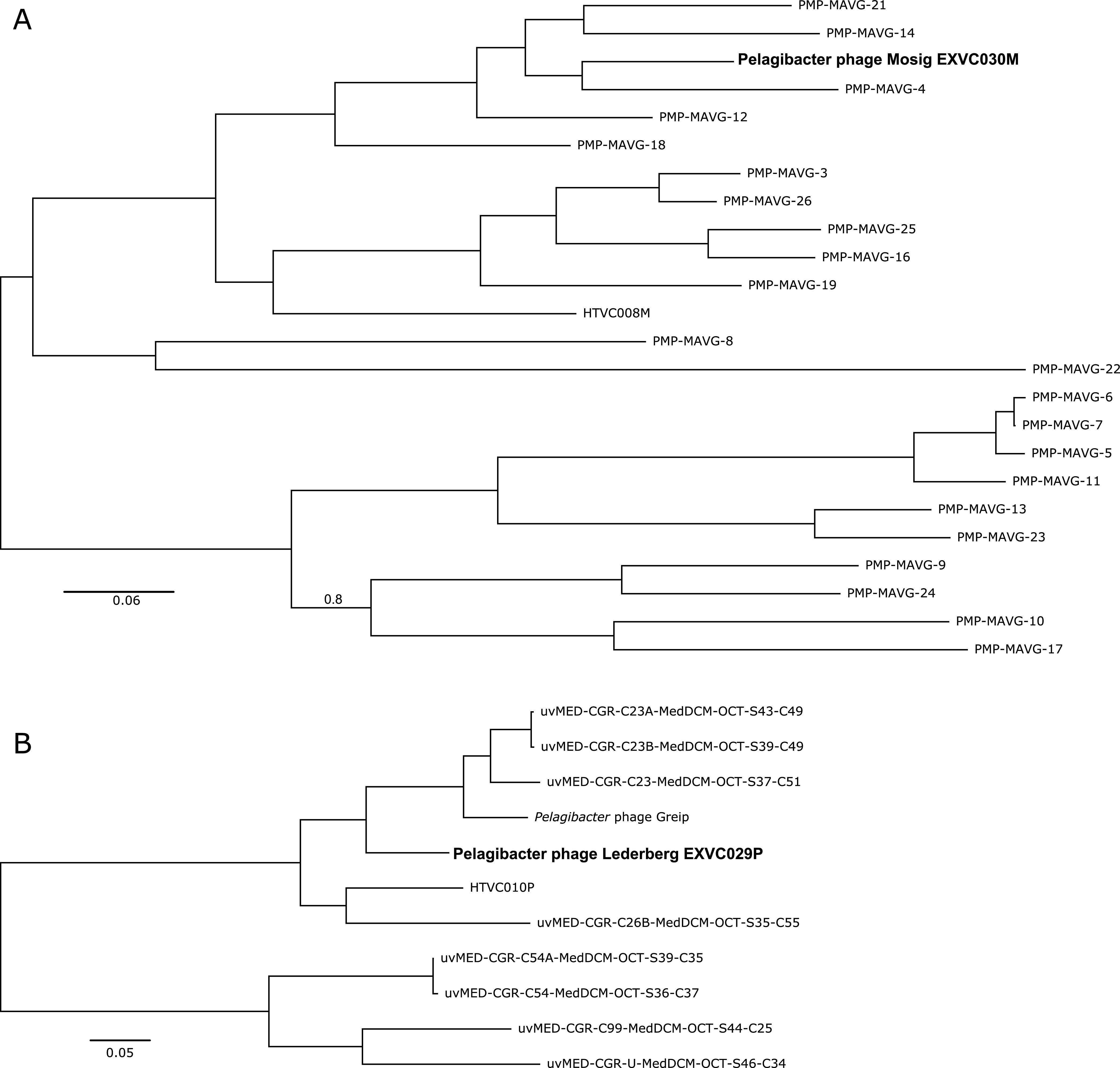
Bayesian inference tree from conserved genes found in pelagiphages (2, 3, 5), contigs from Mediterranean metagenomes (uvMed) (24), and putative pelagimyophages from genome-resolved metagenomics (PMP-MAVG) (25). (A) Terminase large subunit, tail sheath protein, and tail tube protein; (B) head-tail connector protein, capsid assembly protein, major capsid protein, tail tubular protein A, and putative acetyltransferase. Branch support values of 1 were omitted for clarity. The scale bar represents the estimated substitution per site.

DNA libraries (Nextera XT) were prepared and sequenced by the Exeter Sequencing Service (Illumina paired end [2 × 250 bp], NovaSeq S Prime [SP], targeting 30-fold coverage). Raw reads (13.18 million) were trimmed, quality controlled, and error corrected using tadpole (default settings [8] within BBMap v38.22 [https://sourceforge.net/projects/bbmap/]) and assembled with SPAdes v3.13 (7). Viral contigs were confirmed and gene called with VirSorter v1.05 (9) and imported into DNA Master v5.23.3 (10) for manual curation with additional gene calls using GenMark v2.5 (11), GeneMarkS v4.28 (12), GeneMarkS-2 v1.14 (13), GeneMark.hmm v3.25 (14), Glimmer v3.02 (15), and Prodigal v2.6.3 (16). Open reading frames were annotated with NCBI’s nonredundant protein database (17) and phmmer v2.41.1 (18) against the UniProtKB, uniprotrefprot (19), SWISS PROT (20), and Pfam (21) databases (accessed May 2020) and were evaluated using a previously described scoring system (10). Genome completion was verified with CheckV (22). Sequences similar to our isolates were identified with ClusterGenomes v5.1 (https://github.com/simroux/ClusterGenomes) and vConTACT 2 v0.9.19 (23) using previously isolated Pelagiphages (2, 3, 5), fosmid-derived contigs from Mediterranean metagenomes (uvMed) (24), and putative pelagimyophages from genome-resolved metagenomics (PMP-MAVG) (25). Conserved genes were identified (GET_HOMOLOGUES v09072020 [22]), aligned (MUSCLE v3.8.1551 [26]), curated (Gblocks v0.91b [27]), and concatenated manually (all with default settings). Bayesian inference trees were generated via Phylogeny.fr (28) using MRBAYES v3.2.7 (29) (100,000 generations, sampled every 10 generations, 5,000 tree burn-in) (Fig. 1).

Pelagimyophage Mosig (named after microbiologist Gisela Mosig in recognition of her work on Escherichia coli phage T4) was 141,462 bp long (348× coverage; GC content, 30.01%), linear, and 75.73% complete (CheckV [22]). Out of 208 genes, 98 were putative, 3 were tRNAs, 30 were structural, and 77 were associated with DNA replication.

*Pelagibacter* phage Lederberg (named after microbiologist Esther Lederberg in recognition of her work on the E. coli phage λ) was 33,623 bp long (5,849× coverage; GC content, 33.13%) and predicted as circularly permuted/complete. Lederberg had a total of 71 genes, out of which 9 were structural, 8 were associated with DNA replication, and 54 were without known function.

### Data availability.

The complete genome sequences were deposited under GenBank accession numbers MT647605 (Lederberg) and MT647606 (Mosig). The corresponding read data were deposited in the Sequence Read Archive (SRA) under BioProject number PRJNA625644 and SRA accession number SRR12024324.
